# Robotic versus laparoscopic rectal cancer surgery during institutional transition: a 10-year sequential cohort study

**DOI:** 10.3389/fonc.2026.1874238

**Published:** 2026-07-30

**Authors:** Zsolt Madarasz, Krzysztof Nowakowski, Michael Leitz, Bogdan-Cornel Sturzu, Julia Michel, Kira Baginski, Annika Hoyer, Miljana Vladimirov, Jens Hoeppner, Fabian Nimczewski

**Affiliations:** 1 Department of Surgery, Bielefeld University, Medical School and University Medical School Ostwestfalen-Lippe (OWL) – Campus Hospital Lippe, Detmold, Germany; 2 Biostatistics and Medical Biometry, Medical School Ostwestfalen-Lippe (OWL), Bielefeld University, Bielefeld, Germany

**Keywords:** conversion rate, laparoscopic surgery, rectal cancer, robotic surgery, total mesorectal excision (TME)

## Abstract

**Background:**

The potential clinical benefits of robotic rectal cancer surgery compared with conventional laparoscopy remain a matter of debate. This study compared perioperative, pathological, and mid-term oncologic outcomes after robotic and laparoscopic rectal cancer resection in a single-center cohort.

**Methods:**

This retrospective sequential era-based cohort study included consecutive patients with UICC stage I–III rectal adenocarcinoma undergoing elective minimally invasive rectal resection between 2015 and 2024. Laparoscopy represented the institutional standard until June 2018; following implementation of the da Vinci X platform, robotic surgery became the preferred minimally invasive approach. Thus, the present analysis reflects an institutional transition between two surgical eras rather than a contemporaneous comparison of coexisting surgical techniques. The primary outcomes were conversion to open surgery and quality of mesorectal excision. Secondary outcomes included operative duration, hospital stay, anastomotic leakage, major morbidity, reoperation, 30- and 90-day mortality, and oncologic survival endpoints. To reduce baseline imbalance between treatment groups, propensity score overlap weighting was applied.

**Results:**

The study included 239 patients, of whom 146 underwent robotic resection and 93 underwent laparoscopic resection. The rate of conversion to open surgery was significantly lower in the robotic group compared with the laparoscopic group (2.7% vs 15.1%; OR 0.15, 95% CI: [0.02; 0.95], p = 0.044). Mesorectal specimen quality did not differ substantially between groups. Compared with laparoscopy, the robotic approach was associated with a shorter postoperative stay (mean difference −4.72 days, 95% CI: [−6.62; −2.82], p < 0.0001) but a longer operative time (+35.33 min, 95% CI: [17.50; 53.15], p = 0.0001). No statistically significant between-group differences were found for anastomotic leakage, major complications, reoperation, or 30- and 90-day mortality. During follow-up of up to 60 months, no statistically significant differences were observed between groups regarding overall survival, disease-free survival, local recurrence, or distant metastasis.

**Conclusion:**

Within the limitations of this sequential retrospective cohort, robotic rectal cancer surgery was associated with fewer conversions and shorter postoperative hospitalization, without an observed detriment in pathological quality or available mid-term oncologic outcomes. Although these findings suggest potential perioperative advantages of the robotic approach, the study was not powered to establish definitive oncologic equivalence.

## Introduction

Rectal cancer surgery requires precise dissection within a confined pelvic space and remains technically demanding. Complete excision of the mesorectum (TME) is regarded as the key principle for curative rectal cancer surgery. Minimally invasive approaches are now widely applied and have been associated with similar oncologic outcomes and faster postoperative recovery (1, 2). Despite these advances, laparoscopic rectal resection remains technically demanding, particularly in patients with unfavorable pelvic anatomy or bulky distal tumors.

A major limitation of conventional laparoscopy in rectal surgery is the restricted maneuverability of straight instruments within a confined operative field. Accordingly, concerns have been raised not only regarding technical difficulty but also regarding pathological quality parameters such as circumferential resection margin status and completeness of TME (3, 4). Robotic platforms were introduced to address some of these limitations through three-dimensional visualization, articulated instruments, and improved surgeon ergonomics.

Comparative evidence between robotic and laparoscopic rectal cancer surgery remains inconclusive. Early randomized studies did not demonstrate a clear overall advantage for robotic surgery, although subgroup analyses indicated potential advantages in anatomically complex cases (5). More recent studies and meta-analyses have reported lower conversion rates with robotic surgery, whereas differences in postoperative morbidity and long-term oncologic outcomes have generally been modest or inconsistent (6–8). Consequently, the clinical relevance of robotic surgery in routine rectal cancer care remains a matter of ongoing debate.

While randomized trials and recent meta-analyses have provided important comparative evidence between robotic and laparoscopic rectal surgery, real-world implementation data from sequential institutional adoption remain limited. The present study addresses this gap by evaluating a minimally invasive-only, single-center, decade-long consecutive cohort reflecting the institutional transition from laparoscopic to robotic rectal cancer surgery, using propensity score overlap weighting to reduce baseline imbalances. Therefore, the present study evaluates perioperative outcomes, specimen quality, and mid-term oncologic outcomes of robotic versus laparoscopic rectal cancer resection over a 10-year period in a specialized center. We hypothesized that robotic surgery may improve perioperative outcomes without adversely affecting pathological quality or available oncologic outcomes.

## Materials and methods

### Study design and patient selection

We conducted a retrospective sequential era-based cohort study using data from a prospectively maintained institutional database at our colorectal unit. All consecutive patients undergoing elective rectal cancer surgery between January 1, 2015, and December 31, 2024, were screened for eligibility. The preferred minimally invasive technique changed over the study interval as robotic surgery was introduced into routine practice. Between 2015 and mid-2018, rectal resections were performed using a laparoscopic approach. Following the introduction of the da Vinci X system (Intuitive Surgical, Sunnyvale, CA, USA) in June 2018, robotic-assisted surgery became the standard minimally invasive approach for rectal cancer. Thus, the comparison reflects sequential implementation of laparoscopic and robotic approaches over time.

The present study represents a focused reanalysis of the minimally invasive subgroup from our previously published institutional cohort comparing robotic, laparoscopic, and open rectal cancer surgery (9). The prior study included patients treated between 2012 and 2021 and comprised 68 laparoscopic and 62 robotic restorative rectal resections, all of which overlap with the present analysis. In contrast to the previous broader descriptive comparison across all operative approaches, the current study is restricted to minimally invasive procedures and applies propensity score overlap weighting to address the comparative outcomes of robotic versus laparoscopic rectal cancer resection. In addition, the present cohort extends accrual through 2024 and broadens inclusion to all minimally invasive rectal resections irrespective of reconstructive strategy, resulting in an expanded cohort of 93 laparoscopic and 146 robotic procedures. This broader inclusion and updated follow-up form the basis for the present focused propensity-weighted comparative analysis.

**Figure 1:**
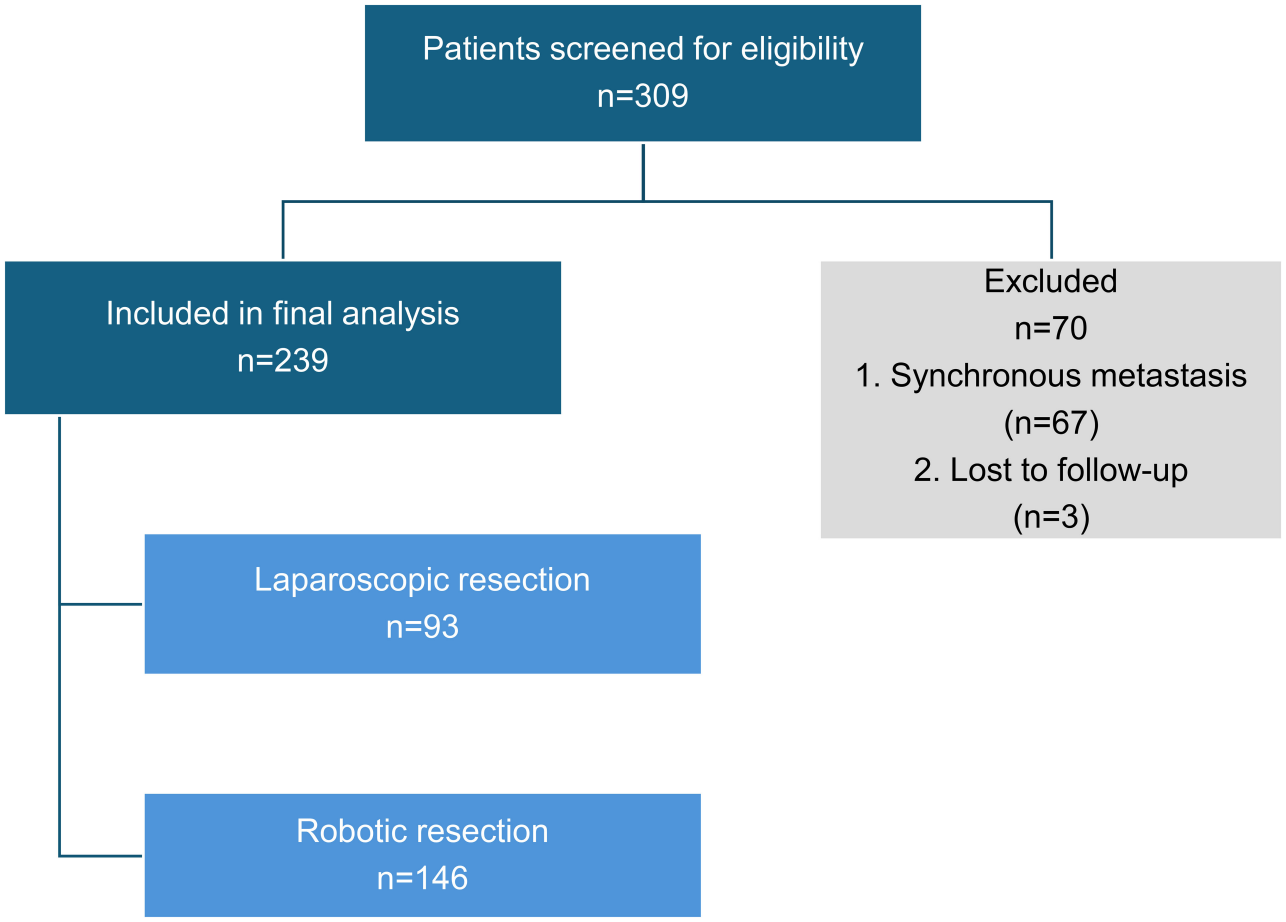
Flowchart of patient selection.

Inclusion criteria were age 18 years or older and histologically proven primary rectal adenocarcinoma with UICC stage I to III disease. Eligible procedures included elective minimally invasive anterior resection with partial mesorectal excision (PME) or total mesorectal excision (TME), Hartmann’s procedure, and abdominoperineal resection (APR). Patients with synchronous metastatic disease (stage IV) or incomplete follow-up were excluded. The patient selection process is illustrated in the study flowchart (**Figure 1**).

The final study cohort comprised 239 patients, including 146 robotic-assisted resections performed using the da Vinci X system and 93 conventional laparoscopic procedures. Because the institutional standard changed completely over time, no parallel allocation or selective assignment to laparoscopic or robotic surgery occurred during the study period.

### Perioperative management and surgical procedure

All procedures were performed by experienced colorectal surgeons with expertise in minimally invasive colorectal surgery. Laparoscopic rectal cancer surgery was first implemented at our institution in 2015, and all consecutive rectal cancer patients were treated laparoscopically thereafter without case selection, thereby inherently including the institutional learning curve for laparoscopic rectal surgery. Following implementation of the da Vinci X platform in June 2018, robotic surgery became the exclusive minimally invasive approach for rectal cancer and likewise included the robotic learning phase without selective patient allocation. Robotic implementation was performed by three colorectal surgeons with established laparoscopic expertise, reflecting the institutional transition from laparoscopic to robotic rectal surgery in routine clinical practice. Accordingly, both treatment groups include the respective implementation phases of each surgical technique, and no procedures were excluded based on surgeon experience in order to reflect real-world practice. Perioperative care was conducted in accordance with the German S3 colorectal cancer guideline applicable during the study period. Treatment decisions, including the indication for neoadjuvant therapy, were made in a multidisciplinary tumor board comprising surgeons, medical and radiation oncologists, radiologists, and pathologists.

During most of the study period, the standard neoadjuvant approach consisted of long-course chemoradiotherapy followed by surgery 6 to 10 weeks after completion of treatment. Total neoadjuvant therapy (TNT) was introduced at our institution in 2022 and was therefore not routinely applied during the majority of the study period.

The surgical technique followed established oncologic principles, including central division of the inferior mesenteric vessels and meticulous dissection along embryological planes. Depending on tumor location, PME or TME was performed. Splenic flexure mobilization was routinely undertaken for low anterior resections with primary anastomosis, whereas it was not mandatory in Hartmann’s procedures or APRs. In restorative resections, colorectal or coloanal anastomoses were usually constructed with a circular stapler. A protective diverting ileostomy was routinely created in patients undergoing low anterior rectal resection. Specimen extraction was routinely performed via a Pfannenstiel incision.

In the robotic group, operations were performed with the da Vinci X robotic system, whereas the laparoscopic group underwent conventional multiport laparoscopy.

From 2016 onward, indocyanine green fluorescence imaging was routinely used intraoperatively to assess perfusion in patients receiving a primary anastomosis. Preoperative bowel preparation and antibiotic prophylaxis were performed according to established institutional protocols.

### Endpoints and follow-up

The primary endpoints were conversion rate and TME quality. Conversion was defined as an unplanned median laparotomy requiring complete abandonment of the minimally invasive approach and transition to an open procedure. Extension of the specimen extraction incision was not classified as conversion. In the present cohort, no emergency conversions due to intraoperative complications occurred. Reasons for conversion included technical difficulties such as challenging splenic flexure mobilization, obesity, or inability to complete pelvic dissection minimally invasively in cases of a narrow pelvis or bulky tumors. Mesorectal specimen quality was assessed by histopathological examination using the Quirke grading system and categorized as complete, nearly complete, or incomplete (10).

Secondary endpoints included length of hospital stay (LOS), anastomotic leakage, operative time, perioperative blood transfusion, major postoperative morbidity according to the Clavien–Dindo classification (≥IIIb) (11), reoperation within 30 days, and 30- and 90-day mortality. Anastomotic leakage was classified according to the International Study Group of Rectal Cancer (ISREC) definition as grade A, B, or C (12). Detailed characteristics and management of all patients with anastomotic leakage are provided in **Supplementary Table 7**.

Patients were followed for up to 60 months following surgery. The median follow-up duration was 52 (Q1–Q3 (44, 60):) months in the robotic group and 60 (Q1–Q3 (60, 60):) months in the laparoscopic group. Follow-up data were obtained from institutional follow-up records, cross-sectional imaging, endoscopic surveillance, multidisciplinary tumor board documentation, and review of electronic medical records. Local recurrence and distant metastasis were additionally analyzed as separate oncologic outcomes. Overall survival (OS) was defined as the interval between surgery and death from any cause. Disease-free survival (DFS) was calculated from the date of surgery to the first occurrence of local recurrence, distant metastasis, or death.

### Statistical analysis

For the statistical analysis, baseline patient characteristics, intraoperative and postoperative outcomes, and histopathological and oncological findings were first summarized descriptively. Continuous variables are summarized as mean values with corresponding standard deviations or median (Q1–Q3), while categorical variables are reported as absolute frequencies and percentages. Median follow-up was estimated using the reverse Kaplan–Meier method.

To address potential confounding due to baseline differences between the robotic surgery (n = 146) and laparoscopic surgery (n = 93) groups, a propensity score (PS) analysis with overlap weights was used. A multivariable logistic regression model was used to derive propensity scores from predefined baseline characteristics. Clinically relevant covariates included in the PS model were age, sex, body mass index (BMI), American Society of Anesthesiologists (ASA) physical status classification, smoking status, previous abdominal surgery, the presence of at least one comorbidity, tumor location (upper rectum, middle rectum, lower rectum), neoadjuvant treatment and procedure (sphincter-preserving procedure, abdominoperineal resection, Hartmann’s procedure). Overlap weights (13) were calculated to emphasize patients with comparable probabilities of receiving either surgery. This method targets the average treatment effect in the overlap population (ATO) and reduces the influence of individuals with extreme propensity scores. After overlap weighting, all covariates included in the propensity score model were well balanced, with weighted absolute standardized mean differences close to zero (see **Supplementary Table 2**; **Supplementary Figure 1**). The association between surgical approach and outcomes was assessed using weighted regression models. For binary outcomes, weighted logistic regression was used. Continuous variables were evaluated using weighted linear regression, and survival outcomes were evaluated using weighted Cox proportional hazards models. TME quality was dichotomized as complete (1) versus non-complete (2-3), combining near complete and incomplete specimens due to the small number of cases in category 3. Weighted Kaplan-Meier curves were generated to visualize overall and disease-free survival across the two surgery groups using overlap weights, with robust sandwich variance estimation. The proportional hazards assumption of both weighted Cox regression models was assessed using Schoenfeld residuals and graphical diagnostics, with no evidence of violation. Results are expressed as mean differences, odds ratios, or hazard ratios with 95% confidence intervals. A complete-case approach was used because there were no missing data for the covariates included in the propensity score model, the study endpoints, or the variables used in the descriptive analyses. All statistical analyses were performed using SAS version 9.4 and R version 4.6.0.

## Results

### Patient characteristics

A total of 239 patients met the inclusion criteria, including 146 patients in the robotic group and 93 in the laparoscopic group. Baseline demographic and clinical characteristics are summarized in **Table 1**. The groups were comparable with regard to age, sex distribution, body mass index, and ASA classification.

**Table 1:** Baseline demographic, clinical, and pre-treatment characteristics.

Characteristic	Robotic group (n=146)	Laparoscopic group (n=93)	Overall (n=239)
Age (in years)			
Mean (SD)	68.3 (12.4)	68.7 (12.3)	68.5 (12.3)
Sex			
Female	49 (33.6%)	41 (44.1%)	90 (37.7%)
Male	97 (66.4%)	52 (55.9%)	149 (62.3%)
BMI (kg/m2)			
Mean (SD)	26.1 (4.18)	26.0 (5.00)	26.1 (4.51)
ASA score			
1	0 (0%)	3 (3.2%)	3 (1.3%)
2	76 (52.1%)	46 (49.5%)	122 (51.0%)
3	66 (45.2%)	41 (44.1%)	107 (44.8%)
4	4 (2.7%)	3 (3.2%)	7 (2.9%)
Comorbidities			
Arterial hypertension	74 (50.7%)	55 (59.1%)	129 (54.0%)
Coronary artery disease	13 (8.9%)	16 (17.2%)	29 (12.1%)
Type II diabetes mellitus	14 (9.6%)	12 (12.9%)	26 (10.9%)
Cardiac insufficiency	15 (10.3%)	10 (10.8%)	25 (10.5%)
Renal insufficiency	4 (2.7%)	1 (1.1%)	5 (2.1%)
Chronic pulmonary disease (COPD)	4 (2.7%)	2 (2.2%)	6 (2.5%)
**Smoking**	29 (19.9%)	20 (21.5%)	49 (20.5%)
Preoperative treatment			
Neoadjuvant treatment	63 (43.2%)	42 (45.2%)	105 (43.9%)
Conventional chemoradiotherapy (CRT)	51 (34.9%)	42 (45.2%)	93 (38.9%)
Total neoadjuvant therapy (TNT)	12 (8.2%)	0 (0%)	12 (5.0%)
**Previous abdominal surgery**	27 (18.5%)	23 (24.7%)	50 (20.9%)
Tumor location			
Upper rectum	30 (20.5%)	20 (21.5%)	50 (20.9%)
Middle rectum	57 (39.0%)	37 (39.8%)	94 (39.3%)
Lower rectum	57 (39.0%)	36 (38.7%)	93 (38.9%)

Neoadjuvant therapy was administered in 43.2% of patients in the robotic group and 45.2% in the laparoscopic group. Tumor location and type of surgical procedure were comparably distributed between groups (**Table 1**).

### Intraoperative outcomes

Intraoperative outcomes are summarized in **Table 2**. The mean operative time was longer in the robotic group (267 ± 74.8 minutes) compared with the laparoscopic group (227 ± 62.6 minutes). Conversion to open surgery occurred less frequently in the robotic group (2.7%) than in the laparoscopic group (15.1%). The distribution of surgical procedures and anastomotic techniques was comparable between groups.

**Table 2:** Intraoperative outcomes.

Outcome	Robotic group (n=146)	Laparoscopic group (n=93)	Overall (n=239)
Operation time (min)			
Mean (SD)	267 (74.8)	227 (62.6)	252 (72.9)
Median [Q1, Q3]	262 (218, 306)	221 (181, 268)	250 (194, 298)
**Conversion to open**	4 (2.7%)	14 (15.1%)	18 (7.5%)
Type of anastomosis			
End-to-End	41 (28.1%)	31 (33.3%)	72 (30.1%)
Side-to-End	64 (43.8%)	28 (30.1%)	92 (38.5%)
Colon J Pouch	2 (1.4%)	7 (7.5%)	9 (3.8%)
Procedure			
Sphincter-Preserving Procedure	107 (73.3%)	59 (63.4%)	166 (69.5%)
Abdominoperineal Resection	27 (18.5%)	22 (23.7%)	49 (20.5%)
Hartmann Procedure	12 (8.2%)	10 (10.8%)	22 (9.2%)

### Postoperative outcomes

Postoperative outcomes are summarized in **Table 3**. Anastomotic leakage was observed in 3 patients (2.1%) in the robotic group and in 8 patients (8.6%) in the laparoscopic group. Most leaks were classified as ISREC grade B and were managed non-operatively with Endo-VAC therapy, drainage, and antibiotics. Two patients required surgical reintervention (grade C). The distribution of leak severity and corresponding treatment strategies is summarized in **Supplementary Table 7**. Major postoperative complications (Clavien–Dindo ≥ IIIb) occurred in 11.6% and 20.4% of patients, respectively. The mean length of hospital stay was shorter in the robotic group (11.8 ± 7.3 days) compared with the laparoscopic group (16.2 ± 8.2 days). Postoperative ICU stay was low in both groups and did not differ substantially, indicating comparable immediate postoperative intensive care requirements. Thirty- and ninety-day postoperative mortality were recorded. Ninety-day mortality occurred in 5 patients (3.4%) in the robotic group and in 2 patients (2.2%) in the laparoscopic group and was identical to 30-day mortality, as no additional deaths occurred between postoperative days 31 and 90. Causes of death were predominantly related to severe cardiopulmonary complications, septic shock, or multiorgan failure, mainly in elderly patients with substantial pre-existing comorbidities. Detailed clinical characteristics and causes of death are summarized in **Supplementary Table 8**.

**Table 3:** Post-operative and short-term outcomes.

Outcome	Robotic group (n=146)	Laparoscopic group (n=93)	Overall (n=239)
Post-operative complications			
Anastomotic leak	3 (2.1%)	8 (8.6%)	11 (4.6%)
Pneumonia	7 (4.8%)	3 (3.2%)	10 (4.2%)
Bladder emptying disorder	0 (0%)	1 (1.1%)	1 (0.4%)
Bowel atony	26 (17.8%)	22 (23.7%)	48 (20.1%)
Urinary tract infection	0 (0%)	1 (1.1%)	1 (0.4%)
Wound infection	10 (6.8%)	9 (9.7%)	19 (7.9%)
Post-operative blood transfusion	1 (0.7%)	4 (4.3%)	5 (2.1%)
Reoperation	15 (10.3%)	19 (20.4%)	34 (14.2%)
**Clavien-Dindo ≥ IIIb**	17 (11.6%)	19 (20.4%)	36 (15.1%)
**90-day mortality**	5 (3.4%)	2 (2.2%)	7 (2.9%)
**30-day readmission**	3 (2.1%)	6 (6.5%)	9 (3.8%)
Length of hospital stay (LOS)			
Mean (SD)	11.8 (7.33)	16.2 (8.24)	13.5 (7.97)
Post-operative intensive care unit (ICU) stay			
Mean (SD)	1.60 (5.35)	2.32 (3.50)	1.88 (4.72)

### Histopathological findings and oncological outcomes

Histopathological characteristics are summarized in **Table 4**. A higher proportion of pT4 tumors was observed in the laparoscopic group. R1 resection occurred in three patients in the laparoscopic group and in none in the robotic group. Circumferential resection margin (CRM) involvement was observed exclusively in the laparoscopic cohort.

**Table 4:** Histopathological and oncological outcomes.

Outcome	Robotic group (n=146)	Laparoscopic group (n=93)	Overall (n=239)
Tumor histology			
Adenocarcinoma	146 (100%)	93 (100%)	239 (100%)
TNM pT stage			
0, pCR	14 (9.6%)	4 (4.3%)	18 (7.5%)
1	24 (16.4%)	9 (9.7%)	33 (13.8%)
2	41 (28.1%)	34 (36.6%)	75 (31.4%)
3	61 (41.8%)	34 (36.6%)	95 (39.7%)
4	6 (4.1%)	12 (12.9%)	18 (7.5%)
TNM pN stage			
0	112 (76.7%)	58 (62.4%)	170 (71.1%)
1	26 (17.8%)	31 (33.3%)	57 (23.8%)
2	8 (5.5%)	4 (4.3%)	12 (5.0%)
TNM pM stage			
0	146 (100%)	93 (100%)	239 (100%)
Lymph nodes retrieved			
Mean (SD)	25.7 (11.9)	28.2 (13.1)	26.7 (12.4)
Lymph nodes infiltrated			
Mean (SD)	0.486 (1.42)	0.742 (1.59)	0.586 (1.49)
Tumor grading			
G1	6 (4.1%)	3 (3.2%)	9 (3.8%)
G2	135 (92.5%)	87 (93.5%)	222 (92.9%)
G3	5 (3.4%)	3 (3.2%)	8 (3.3%)
**Tumor deposits (TD)**	10 (6.8%)	20 (21.5%)	30 (12.6%)
**Perineural invasion (Pn)**	7 (4.8%)	10 (10.8%)	17 (7.1%)
**Lymphovascular invasion (L)**	21 (14.4%)	27 (29.0%)	48 (20.1%)
**Vascular invasion (V)**	5 (3.4%)	11 (11.8%)	16 (6.7%)
Resection status			
0	146 (100%)	90 (96.8%)	236 (98.7%)
1	0 (0%)	3 (3.2%)	3 (1.3%)
CRM status			
CRM negative	146 (100%)	90 (96.8%)	236 (98.7%)
CRM positive	0 (0%)	3 (3.2%)	3 (1.3%)
TME quality			
1 (complete)	132 (90.4%)	79 (84.9%)	211 (88.3%)
2 (near complete)	11 (7.5%)	6 (6.5%)	17 (7.1%)
3 (incomplete)	3 (2.1%)	8 (8.6%)	11 (4.6%)
UICC Stage			
0	13 (8.9%)	4 (4.3%)	17 (7.1%)
I	55 (37.7%)	35 (37.6%)	90 (37.7%)
II	44 (30.1%)	17 (18.3%)	61 (25.5%)
III	34 (23.3%)	37 (39.8%)	71 (29.7%)
Mid-Term Oncologic Outcomes			
Local recurrence	4 (2.7%)	8 (8.6%)	12 (5.0%)
Distant metastasis	23 (15.8%)	16 (17.2%)	39 (16.3%)

### Mid-term oncologic outcomes and regression analyses

**Figure 2:**
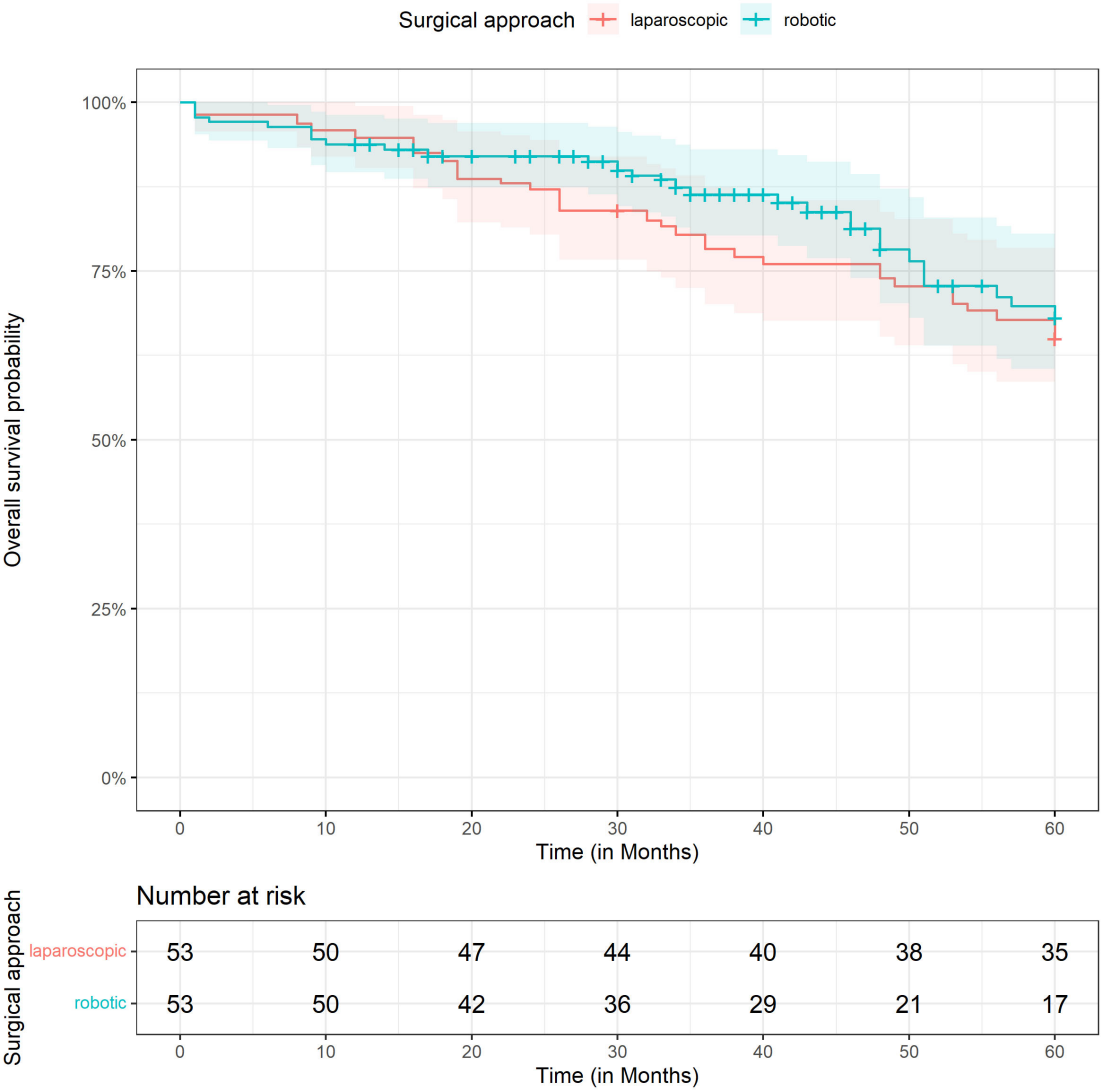
Kaplan-Meier curve for overall survival with overlap weights.

**Figure 3:**
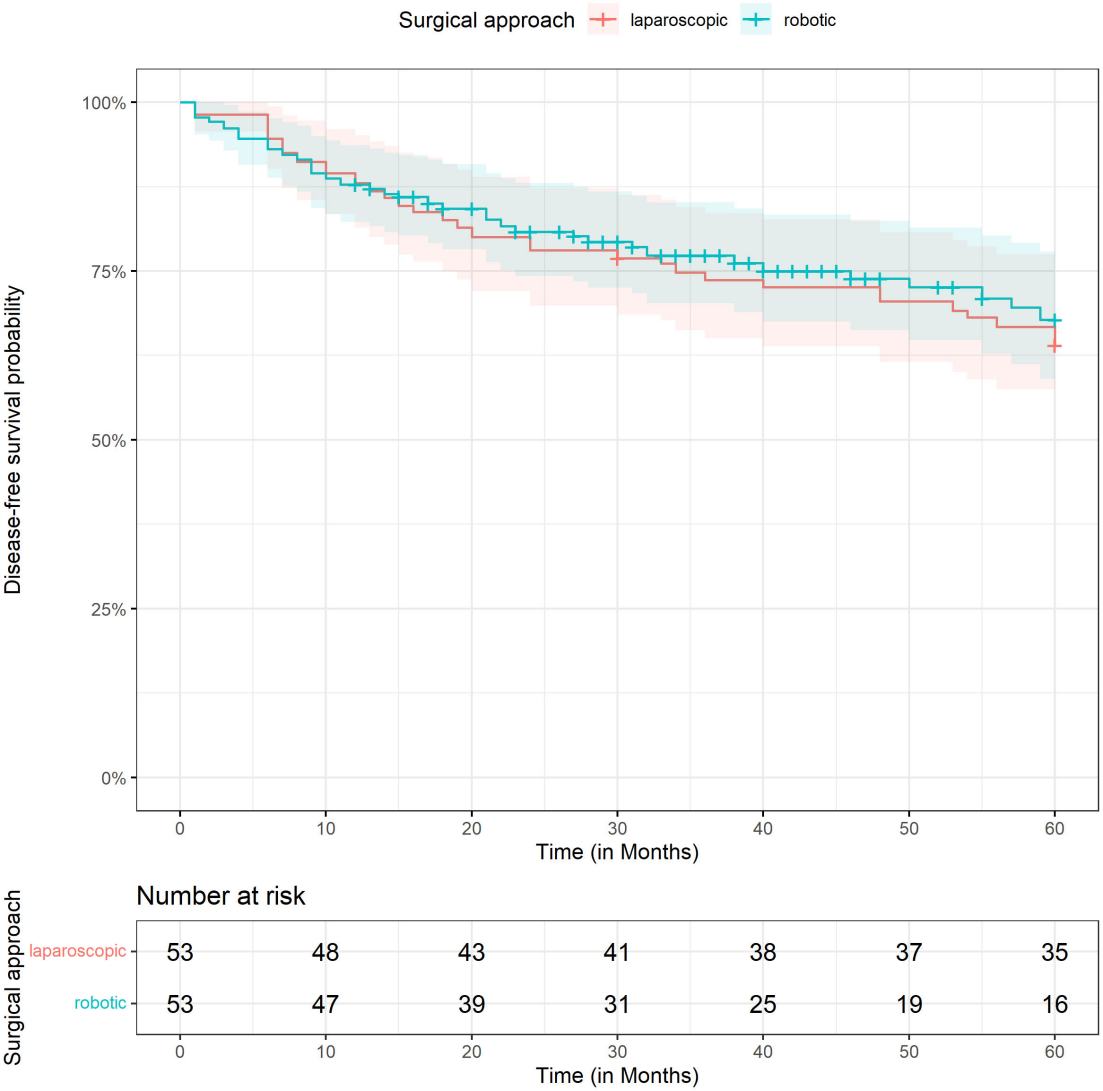
Kaplan-Meier curve for disease-free survival with overlap weights.

Mid-term cancer-related outcomes are summarized in **Table 4**, whereas results from weighted regression analyses are presented in **Table 5**. Overall survival (**Figure 2**) and disease-free survival (**Figure 3**) did not differ significantly between groups. No statistically significant association was observed between surgical approach and either overall survival (HR 0.84, 95% CI: [0.51; 1.37], p = 0.482) or disease-free survival (HR 0.89, 95% CI: [0.56; 1.42], p = 0.629). Detailed numbers at risk, events, and survival probabilities over time for overall survival and disease-free survival, stratified by treatment group, are provided in **Supplementary Tables 3**–**S6**, for both weighted and unweighted analyses.

**Table 5:** Multivariable regression analyses.

Outcome	Hazard ratio*	95% CI^1^	p-value
**Overall survival**	0.84	[0.51; 1.37]	0.4821
**Disease-free survival**	0.89	[0.56; 1.42]	0.6294
	Mean difference*	95% CI^1^	
**Length of hospital stay (LOS)**	-4.72	[-6.62; -2.82]	<.0001
**Operation time (min)**	35.33	[17.50; 53.15]	0.0001
	Odds ratio*	95% CI^1^	
**Conversion**	0.15	[0.02; 0.95]	0.0441
**TME quality**	0.59	[0.19; 1.86]	0.3710
**Anastomotic leak**	0.19	[0.02; 1.62]	0.1283
**Clavien-Dindo ≥ IIIb**	0.48	[0.16; 1.43]	0.1869
**Reoperation**	0.44	[0.15; 1.33]	0.1456
**90-day mortality**	1.59	[0.12; 21.15]	0.7255
**Postoperative blood transfusion**	0.12	[0.01; 7.32]	0.3128
**Local recurrence during follow-up**	0.31	[0.05; 1.94]	0.2082
**Metastasis during follow-up**	0.96	[0.35; 2.64]	0.9392

^1^ Confidence Interval, *Robotic vs. Laparoscopic.

Local recurrence occurred in 4 patients (2.7%) in the robotic group and 8 patients (8.6%) in the laparoscopic group. Distant metastases were observed in 23 (15.8%) and 16 (17.2%) patients, respectively.

In the propensity score weighted regression analysis, robotic surgery was associated with a significantly lower risk of conversion to open surgery (OR 0.15, 95% CI: [0.02; 0.95], p = 0.044). Robotic surgery was also associated with a significantly shorter postoperative stay (mean difference −4.72 days, 95% CI: [−6.62; −2.82], p < 0.0001), whereas operative duration was significantly longer in the robotic group, with a mean increase of 35.33 minutes (95% CI: [17.50; 53.15], p = 0.0001).

No statistically significant associations were observed between surgical approach and TME quality, anastomotic leakage, major postoperative complications (Clavien–Dindo ≥ IIIb), reoperation, postoperative blood transfusion, local recurrence, or distant metastasis (**Table 5**).

## Discussion

### Principal findings

The present 10-year single-center cohort analysis demonstrates that robotic rectal cancer surgery was associated with a lower rate of conversion to open surgery and a shorter postoperative hospital stay compared with laparoscopic surgery. Importantly, no significant differences were observed between the two approaches regarding oncologic quality parameters or mid-term survival outcomes. The present findings should be interpreted in the context of a sequential implementation design rather than as an isolated platform comparison. Because robotic surgery replaced laparoscopy as the institutional standard after 2018, temporal changes in perioperative pathways, discharge management, neoadjuvant treatment strategies, and overall institutional experience may have contributed to the observed differences. Notably, the longer operative time observed in the robotic group did not translate into increased postoperative morbidity. The present results suggest that robotic instrumentation may facilitate pelvic dissection and contribute to favorable perioperative outcomes while preserving acceptable oncologic quality parameters. However, no conclusions regarding functional outcomes such as urinary, sexual, or bowel function can be drawn from the present analysis. Similar findings have been described in randomized studies and comparative analyses evaluating robotic and laparoscopic rectal cancer surgery (14–17).

### Conversion to open surgery

The need to convert to an open procedure is often interpreted as a surrogate of intraoperative technical difficulty. Earlier studies suggest that conversion to open surgery is linked to increased postoperative morbidity and longer recovery. Previous pooled analyses examining conversion during laparoscopic colorectal surgery reported significantly worse postoperative outcomes in converted cases (18). In addition, long-term follow-up analyses have suggested that conversion may adversely influence oncologic results after laparoscopic colorectal resections (19).

In the present cohort, robotic surgery was associated with lower odds of conversion compared with laparoscopic surgery (OR 0.15), corresponding to an approximately 84% reduction in the odds of conversion. Importantly, the lower conversion rate was observed despite inclusion of the robotic implementation phase, suggesting that this advantage persisted even during early institutional adoption. Similar findings have been reported in large real-world institutional cohorts, demonstrating lower conversion rates and favorable perioperative outcomes with robotic rectal cancer surgery compared with laparoscopy (20). A national cohort analysis demonstrated that robotic rectal surgery was associated with significantly fewer conversions compared with conventional laparoscopy (21). Likewise, population-level data from the Dutch Surgical Colorectal Audit confirmed that conversion remains a relevant challenge in laparoscopic colorectal cancer surgery (22).

The lower conversion rate observed with robotic surgery may be explained by technical characteristics of the robotic system, including enhanced visualization, wristed instrumentation, and improved surgeon ergonomics. These features may be particularly advantageous in technically demanding situations such as narrow male pelvis anatomy, obesity, or tumors located in the distal rectum.

### Perioperative outcomes

Despite longer operative duration, robotic surgery was not associated with increased postoperative morbidity in the present study. However, patients undergoing robotic surgery experienced a shorter hospital stay. A meta-analysis of randomized trials evaluating robotic and laparoscopic rectal surgery reported lower conversion rates and comparable complication profiles between both techniques (17). Similarly, a systematic review focusing on patients undergoing TME following neoadjuvant chemoradiotherapy demonstrated comparable perioperative morbidity between robotic and laparoscopic procedures while highlighting technical advantages of the robotic platform (23). Additional meta-analyses evaluating postoperative complications have likewise reported similar overall morbidity between both approaches (24). Comparable short-term perioperative findings have also been reported in real-world non-randomized cohorts, supporting the external validity of the present observations in routine colorectal practice (25).

### Oncologic outcomes

Ensuring adequate oncologic resection remains a key requirement when assessing novel surgical approaches in rectal cancer. In the present study, key oncologic surrogate markers, including TME quality and resection margin status, did not differ significantly between robotic and laparoscopic surgery. Furthermore, no significant differences were observed in overall survival, disease-free survival, local recurrence, or distant metastasis between both approaches. However, the present study may be underpowered to detect smaller but potentially clinically relevant differences in oncologic endpoints, particularly regarding local recurrence, CRM positivity, and TME completeness.

These findings align with previously published comparative studies investigating oncologic outcomes after robotic rectal cancer surgery. A comparative analysis demonstrated similar survival outcomes following robotic and laparoscopic low anterior resection (15). In addition, a large European multicenter propensity score-matched analysis comparing robotic, laparoscopic, and transanal TME reported comparable oncologic results between minimally invasive techniques (26).

More recent analyses further support these observations. A propensity score-matched study reported comparable perioperative and oncologic outcomes between robotic and laparoscopic rectal cancer surgery (27). Similarly, a meta-analysis of randomized controlled trials demonstrated no significant differences in overall or disease-free survival between both surgical approaches (28). Additional systematic reviews have likewise reported similar oncologic outcomes between robotic and laparoscopic rectal surgery (16, 29).

### Strengths of the study

The present study has several strengths. First, it reflects routine clinical practice over an extended observation period of ten years within a specialized colorectal center. Second, the inclusion of consecutive patients minimizes the likelihood of selection bias. Third, the application of propensity score overlap weighting allowed adjustment for baseline differences between treatment groups and improved comparability of the analyzed cohorts. Moreover, this focused minimally invasive reanalysis extends our previously published institutional cohort and provides an updated propensity-weighted comparative assessment of robotic and laparoscopic rectal cancer surgery.

### Limitations

This study has several limitations. First, its retrospective design may introduce residual confounding despite statistical adjustment. In addition, robotic surgery was introduced in 2018, resulting in a sequential era-based cohort design. Consequently, the comparison reflects not only differences between surgical platforms but also temporal changes in institutional practice. During the study period, perioperative care pathways, discharge protocols, neoadjuvant treatment strategies, and surgeon experience may have evolved and could have influenced the observed outcomes independently of surgical technique. Furthermore, both the laparoscopic and robotic cohorts included their respective implementation phases, and learning-curve effects may therefore have contributed to perioperative differences. Notably, despite inclusion of the robotic learning phase, conversion rates remained significantly lower in the robotic cohort. In particular, the shorter postoperative hospital stay observed in the robotic cohort may partly reflect organizational developments over time rather than the surgical platform alone. Although no formal patient selection for surgical technique was performed, residual confounding due to unmeasured era-related factors cannot be excluded. Furthermore, functional outcomes, including urinary, sexual, and bowel function, were not evaluated in the present analysis. This represents an important limitation, as potential functional benefits are frequently discussed in the context of robotic rectal surgery but cannot be assessed based on the current data. Cost-effectiveness was likewise not evaluated. Despite these limitations, the study provides real-world data from a high-volume colorectal center over a prolonged observation period.

## Conclusion

Within the limitations of this sequential retrospective cohort, robotic rectal cancer surgery was associated with lower conversion rates and shorter postoperative hospital stay compared with laparoscopic rectal cancer surgery. Despite longer operative times, no apparent detriment was observed regarding pathological quality or available mid-term oncologic outcomes. These findings should be interpreted in the context of a sequential institutional transition and do not establish definitive oncologic equivalence.

## Data Availability

The original contributions presented in the study are included in the article/**Supplementary Material**. Further inquiries can be directed to the corresponding author.
