# Supplementary material for: Robotic versus laparoscopic rectal cancer surgery during institutional transition: a 10-year sequential cohort study
Source: Front Oncol. 2026 Jul 30;16:1874238. doi: 10.3389/fonc.2026.1874238 (PMC13467983; doi:10.3389/fonc.2026.1874238)
Supplement: Supplementary file 1 [file DataSheet1.pdf]

Supplementary Material to

Robotic versus Laparoscopic Rectal Cancer Surgery During Institutional Transition: A 10-Year Sequential Cohort Study

Authors: Zsolt Madarasz, Krzysztof Nowakowski, Michael Leitz, Bogdan-Cornel Sturzu, Julia Michel, Kira Baginski, Annika Hoyer, Miljana Vladimirov, Jens Hoeppner, Fabian Nimczewski

Table S1: Estimation specifications

**1. Step: Estimation of Propensity Score with overlap weights**				
Model	*Outcome*	*Included covariates*	*Link function*	*Weights*
Logistic regression	$P(Y=1)$:<br>Probability of receiving robot-assisted surgery<br><br>Propensity score defined as:<br><br>$e(X) = P(Y = 1 \vee X)$	age, sex, body mass index (BMI), American Society of Anesthesiologists (ASA) physical status classification, smoking status, previous abdominal surgery, the presence of at least one comorbidity, tumor location (upper rectum, middle rectum, lower rectum), neoadjuvant treatment, and type of procedure (sphincter-preserving procedure, abdominoperineal resection, Hartmann procedure).	Logit<br>$logit\{P(Y=1\|X)\} = log\left(\frac{P(Y = 1 \vee X)}{1 - P(Y = 1 \vee X)}\right)$	Overlap weights:<br>$w_i = \begin{cases} 1 - e(X_i), if Y_i = 1 \\ e(X_i), if Y_i = 0 \end{cases}$
**2. Step: Modelling primary and secondary endpoints using a PS-model with overlap weights**				
Weighted logistic regression	<u>Binary outcomes:</u><br>Conversion, TME quality, anastomotic leak, Clavien-Dindo≥ IIIb, reoperation, 30-day mortality, postoperative blood transfusion, local recurrence during follow-up, metastasis during follow-up	Surgical approach (robot-assisted vs. laparoscopic surgery)	<u>Link function:</u><br>logit<br><br><u>Estimation:</u><br>Maximum likelihood estimation using Fisher scoring algorithm	Overlap weights
Weighted linear regression	<u>Continuous outcomes:</u><br>Length of hospital stay (LOS), Operation time (min)	Surgical approach (robot-assisted vs. laparoscopic surgery)	<u>Link function:</u><br>Identity<br><br><u>Estimation:</u><br>Weighted least squares (equivalently GLS with diagonal weight matrix defined by overlap weights)	Overlap weights

Weighted Cox proportional hazards model	Time-to-event outcomes:<br>Overall survival, Disease-free survival	Surgical approach (robot-assisted vs. laparoscopic surgery)	Model specification:<br>Cox proportional hazards model<br><br>Estimation:<br>Partial likelihood estimation<br><br>Handling of ties:<br>Breslow method	Overlap weights

Table S2: Standardized mean differences before and after propensity score overlap weighting

Variable	Standardized Mean Difference (Unweighted)	Standardized Mean Difference (Weighted)
**Age (in years)**	-0.036525794	-0.00000137195647
**Sex**	0.105243777	0.00000047424603
**BMI (kg/m2)**	0.002046113	-0.00000005644163
**ASA score**		
1	-0.032258065	-0.00000074229544
2	0.025924289	0.00000119543786
3	0.011194579	-0.00000037381062
4	-0.004860804	-0.00000007933180
**Smoking**	-0.016423626	-0.00000027694386
**Previous abdominal surgery**	-0.062380321	-0.00000050726701
**Presence of at least one comorbidity**	-0.066872883	-0.00000035381007
**Tumor location**		
Lower rectum	0.003314185	-0.00000011645794
Middle rectum	-0.007438503	0.00000046893574
Upper rectum	-0.009574311	-0.00000085203089
**Procedure**		
Hartmann Procedure	-0.025335101	-0.00000021662811
Abdominoperineal Resection	-0.051627633	0.00000041677569
Sphincter-Preserving Procedure	0.098468110	0.00000028680058
**Neoadjuvant treatment**	-0.020106054	0.00000165021616

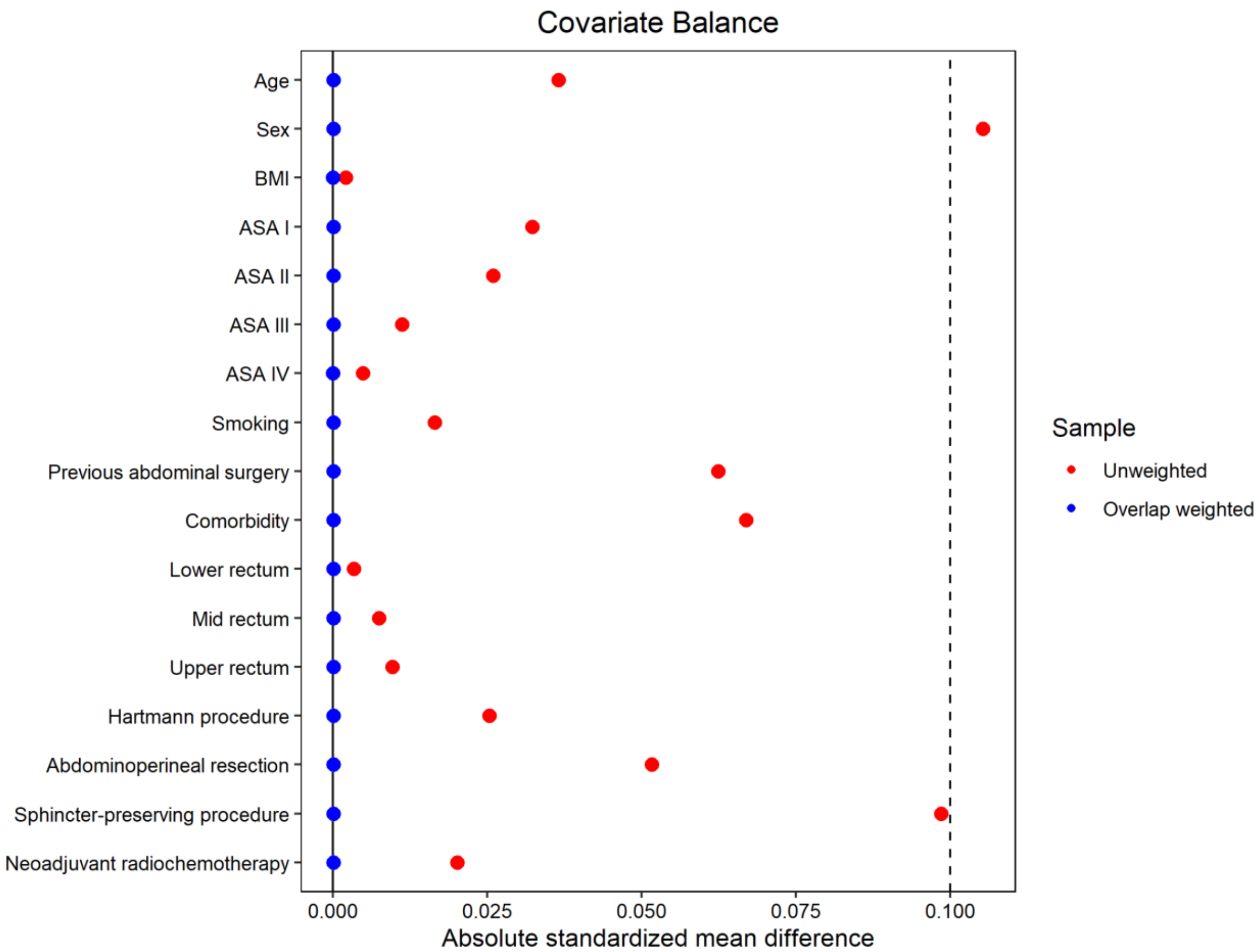

Figure S1: Covariate balance before and after overlap weighting

Table S3: Weighted Kaplan–Meier estimates of overall survival using overlap weighting, including numbers at risk, events, and censoring over time

Time (Months)	Group	Number at risk	Events	Cumulative Events	Censored	Cumulative Censored	Survival probability (%)	95%-Confidence interval (%)
0	laparoscopic	52.61174	0.0000000	0.0000000	0.0000000	0.0000000	100	[100; 100]
5		51.65214	0.9596073	0.9596073	0.0000000	0.0000000	98	[96; 100]
10		50.45725	1.1948855	2.1544928	0.0000000	0.0000000	96	[92; 100]
15		49.83762	0.6196310	2.7741237	0.0000000	0.0000000	95	[90; 99]
20		46.65794	3.1796764	5.9538002	0.0000000	0.0000000	89	[82; 96]
25		45.83020	0.8277422	6.7815424	0.0000000	0.0000000	87	[80; 94]
30		44.16474	1.6654629	8.4470052	0.5900475	0.5900475	84	[77; 92]
35		41.72240	1.8522925	10.2992978	0.0000000	0.5900475	80	[72; 89]
40		40.02666	2.2432889	12.5425867	0.0000000	0.5900475	76	[68; 86]
45		39.47911	0.0000000	12.5425867	0.0000000	0.5900475	76	[68; 86]
50		37.76066	1.7184526	14.2610393	0.0000000	0.5900475	73	[64; 83]
55		35.90267	1.8579902	16.1190295	0.0000000	0.5900475	69	[60; 80]
60		35.17942	2.1552505	18.2742800	33.7474170	34.3374645	65	[56; 76]
0	robotic	52.61171	0.0000000	0.0000000	0.0000000	0.0000000	100	[100; 100]
5		51.10236	1.5093484	1.5093484	0.0000000	0.0000000	97	[94; 100]
10		49.75150	1.7685092	3.2778575	0.0000000	0.0000000	94	[90; 98]
15		47.28425	0.4013787	3.6792362	2.4454273	2.4454273	93	[89; 98]
20		42.38970	0.4702588	4.1494951	4.5262500	6.9716773	92	[87; 97]
25		39.28880	0.0000000	4.1494951	2.2017400	9.1734173	92	[87; 97]
30		36.14417	0.8552755	5.0047706	3.0683003	12.2417175	90	[85; 96]
35		32.22033	1.3495109	6.3542816	3.0300886	15.2718061	86	[80; 93]
40		29.06165	0.0000000	6.3542816	2.3491131	17.6209192	86	[80; 93]
45		25.26471	0.8630610	7.2173425	2.9980321	20.6189513	84	[77; 91]
50		20.98493	2.0397510	9.2570936	2.2312721	22.8502234	76	[68; 86]
55		18.37682	0.9829120	10.2400056	1.8671194	24.7173428	73	[64; 83]
60		16.92696	1.1548473	11.3948529	16.4995121	41.2168549	68	[58; 79]

Table S4: Kaplan–Meier estimates of overall survival without overlap weighting (unweighted), including numbers at risk, events, and censoring over time

Time (Months)	Group	Number at risk	Events	Cumulative Events	Censored	Cumulative Censored	Survival probability (%)	95%-Confidence interval (%)
0	laparoscopic	93	0	0	0	0	100	[100; 100]
5		91	2	2	0	0	98	[95; 100]
10		89	2	4	0	0	96	[92; 100]
15		88	1	5	0	0	95	[90; 99]
20		83	5	10	0	0	89	[83; 96]
25		81	2	12	0	0	87	[81; 94]
30		78	3	15	1	1	84	[77; 92]
35		74	3	18	0	1	81	[73; 89]
40		70	5	23	0	1	75	[67; 84]
45		69	0	23	0	1	75	[67; 84]
50		66	3	26	0	1	72	[63; 82]
55		63	3	29	0	1	69	[60; 79]
60		62	3	32	60	61	65	[56; 76]
0	robotic	146	0	0	0	0	100	[100; 100]
5		141	5	5	0	0	97	[94; 100]
10		138	4	9	0	0	94	[90; 98]
15		132	1	10	6	6	93	[89; 97]
20		120	1	11	11	17	92	[88; 97]
25		112	0	11	6	23	92	[88; 97]
30		103	2	13	9	32	91	[86; 96]
35		91	5	18	8	40	86	[80; 92]
40		82	0	18	7	47	86	[80; 92]
45		70	2	20	10	57	84	[78; 91]
50		58	5	25	7	64	77	[70; 86]
55		51	3	28	5	69	73	[65; 83]
60		47	3	31	46	115	69	[60; 79]

Table S5: Weighted Kaplan–Meier estimates of disease-free survival using overlap weighting, including numbers at risk, events, and censoring over time

Time (Months)	Group	Number at risk	Events	Cumulative Events	Censored	Cumulative Censored	Survival probability (%)	95%-Confidence interval (%)
0	laparoscopic	52.61174	0.0000000	0.0000000	0.0000000	0.0000000	100	[100; 100]
5		51.65214	0.9596073	0.9596073	0.0000000	0.0000000	98	[96; 100]
10		47.98008	4.5500247	5.5096320	0.0000000	0.0000000	90	[83; 96]
15		45.18210	2.5670034	8.0766354	0.0000000	0.0000000	85	[77; 93]
20		42.86759	2.4200571	10.4966924	0.0000000	0.0000000	80	[72; 89]
25		41.08774	1.0273154	11.5240078	0.0000000	0.0000000	78	[70; 87]
30		41.08774	0.6180339	12.1420417	0.5900475	0.5900475	77	[69; 86]
35		38.77990	1.0997559	13.2417976	0.0000000	0.5900475	75	[66; 84]
40		38.21135	1.1160993	14.3578969	0.0000000	0.5900475	73	[64; 83]
45		37.66380	0.0000000	14.3578969	0.0000000	0.5900475	73	[64; 83]
50		36.56338	1.1004187	15.4583156	0.0000000	0.5900475	71	[62; 81]
55		35.32074	1.2426450	16.7009606	0.0000000	0.5900475	68	[59; 79]
60		34.59749	2.1552505	18.8562111	33.1654859	33.7555334	64	[55; 75]
0	robotic	52.61171	0.0000000	0.0000000	0.0000000	0.0000000	100	[100; 100]
5		49.76523	2.8464788	2.8464788	0.0000000	0.0000000	95	[91; 99]
10		47.08154	3.1013384	5.9478172	0.0000000	0.0000000	89	[83; 94]
15		43.84075	1.3881776	7.3359948	2.4454273	2.4454273	86	[80; 92]
20		38.68622	0.8400239	8.1760187	4.2032073	6.6486346	84	[78; 91]
25		34.03638	1.5489382	9.7249569	2.2017400	8.8503747	81	[74; 88]
30		30.98575	0.6153455	10.3403024	2.6985352	11.5489099	79	[73; 87]
35		28.45605	0.7730899	11.1133923	2.3608761	13.9097860	77	[70; 85]
40		25.29154	0.7764164	11.8898088	2.3491131	16.2588991	75	[68; 83]
45		22.42490	0.0000000	11.8898088	2.5273910	18.7862900	75	[68; 83]
50		19.37918	0.6592873	12.5490960	2.2312721	21.0175621	73	[65; 81]
55		17.90039	0.4073922	12.9564883	1.8671194	22.8846815	71	[63; 80]
60		16.02308	0.7474551	13.7039433	16.0230830	38.9077644	68	[59; 78]

Table S6: Kaplan–Meier estimates of disease-free survival without overlap weighting (unweighted), including numbers at risk, events, and censoring over time

Time (Months)	Group	Number at risk	Events	Cumulative Events	Censored	Cumulative Censored	Survival probability (%)	95%-Confidence interval (%)
0	laparoscopic	93	0	0	0	0	100	[100; 100]
5		91	2	2	0	0	98	[95; 100]
10		85	8	10	0	0	89	[83; 96]
15		80	4	14	0	0	85	[78; 93]
20		76	4	18	0	0	81	[73; 89]
25		73	2	20	0	0	78	[71; 87]
30		73	1	21	1	1	77	[69; 86]
35		69	2	23	0	1	75	[67; 85]
40		67	3	26	0	1	72	[63; 82]
45		66	0	26	0	1	72	[63; 82]
50		64	2	28	0	1	70	[61; 80]
55		62	2	30	0	1	68	[59; 78]
60		61	3	33	59	60	64	[55; 75]
0	robotic	146	0	0	0	0	100	[100; 100]
5		138	8	8	0	0	95	[91; 98]
10		131	8	16	0	0	89	[84; 94]
15		123	4	20	6	6	86	[81; 92]
20		110	2	22	10	16	85	[79; 91]
25		98	4	26	6	22	82	[75; 88]
30		89	2	28	8	30	80	[74; 87]
35		81	3	31	6	36	77	[70; 85]
40		72	2	33	7	43	75	[68; 83]
45		63	0	33	8	51	75	[68; 83]
50		54	2	35	7	58	73	[65; 81]
55		50	1	36	5	63	71	[63; 80]
60		45	2	38	45	108	68	[60; 78]

Table S7. Clinical characteristics and management of patients with anastomotic leakage

Patient	App.	Year	Age	Sex	Tumor height (cm)	Neo-adj.	Presacral Abscess	Management	ISREC grade
1	Lap	2015	50	M	5	Yes	Yes	Endo-VAC + antibiotics	B
2	Lap	2015	58	F	9	Yes	Yes	CT-guided drainage + Endo-VAC + antibiotics	B
3	Lap	2015	54	F	7	Yes	Yes	Endo-VAC + antibiotics	B
4	Lap	2016	53	M	9	Yes	No	Endo-VAC + antibiotics	B
5	Lap	2016	55	M	10	Yes	Yes	CT-guided drainage + Endo-VAC + antibiotics	B
6	Lap	2016	64	M	5	Yes	Yes	Relaparoscopy + drainage + antibiotics	C
7	Lap	2017	75	M	9	No	Yes	CT-guided drainage + antibiotics	B
8	Lap	2017	70	M	7	Yes	No	Endo-VAC + antibiotics	B
9	Rob	2019	78	F	5	Yes	No	Endo-VAC + antibiotics	B
10	Rob	2020	84	F	10	No	Yes	Relaparoscopy + rectal extirpation + end colostomy + antibiotics	C
11	Rob	2021	29	M	12	No	No	Endo-VAC + antibiotics	B

Abbreviations: App. = Approach; M= male; F= female; Lap = laparoscopic; Rob = robotic; Neo-adj. = neoadjuvant therapy; ISREC = International Study Group of Rectal Cancer.

Anastomotic leakage was classified according to the International Study Group of Rectal Cancer (ISREC). Grade B leaks required active therapeutic intervention without relaparotomy, whereas grade C leaks required surgical reintervention.

Weitz, J., Büchler, M., Rahbari, N., Welsch, T., Contin, P., Bruckner, T., Ulrich, A., & Kulu, Y. (2013). Validation of the International Study Group of Rectal Cancer definition and severity grading of anastomotic leakage. Surgery, 153(6), 753–761. https://doi.org/10.1016/j.surg.2013.02.007

Table S8. Clinical characteristics and causes of postoperative mortality (90-day mortality)

Patient	Approach	Age	Sex	ASA	Cause of death
1	Lap Hartmann's procedure	79	female	3	Septic complications + respiratory failure
2	Lap Hartmann's procedure	88	male	4	Cardiopulmonary decompensation
3	Rob low anterior resection	80	male	3	Respiratory/cardiac failure
4	Rob Hartmann's procedure	78	male	3	Aspiration pneumonia + sepsis/MOF
5	Rob low anterior resection	70	male	3	Suspected PE/cardiopulmonary arrest
6	Rob Hartmann's procedure	84	female	3	Bowel ischemia + septic shock
7	Rob Hartmann's procedure	73	male	3	Septic shock + bacteremia

Abbreviations: Lap = laparoscopic; Rob = robotic; MOF = multiorgan failure; PE = pulmonary embolism

Ninety-day mortality was identical to 30-day mortality in both groups, as no additional deaths occurred between postoperative day 31 and 90. Causes of death were predominantly related to severe cardiopulmonary complications, septic shock, or multiorgan failure, frequently in elderly patients with substantial pre-existing comorbidities.
